# Cervical ectopic pregnancy – the first case of live birth and uterus-conserving management

**DOI:** 10.1186/s12884-023-05951-5

**Published:** 2023-09-15

**Authors:** Angela Köninger, Buu-Phuc Nguyen, Udo Schwenk, Mehmet Vural, Antonella Iannaccone, Jens Theysohn, Rainer Kimmig

**Affiliations:** 1 Department of Gynecology and Obstetrics, University Hospital Essen, Hufelandstrasse 55, Essen, 45147 Germany; 2 Department of Gynecology and Obstetrics, Hospital St. Hedwig of the Order of St. John, University of Regensburg, Steinmetzstrasse 1-3, 93049 Regensburg, Germany; 3 Institute of Diagnostic and Interventional Radiology and Neuroradiology, University Hospital Essen, Hufelandstrasse 55, Essen, 45147 Germany

**Keywords:** Cervical ectopic pregnancy, Live birth, Uterus-conservation, Uterine artery embolization, Cervical internal os plasty

## Abstract

A 37-old III gravida II para with two previous cesarean sections (CS) presented in 7 + 3 weeks of pregnancy with cervical ectopic pregnancy (CEP). At 12th week of pregnancy, a cerclage was performed to avoid cervical distention by the expanding placenta. Due to missing experience in CEP management and to avoid emergency operation, we recommended CS in 30th week of pregnancy due to unspecific pain of the patient. Vaginal bleeding never occured.

After transverse laparotomy, the urinary bladder was sharply dissected from the anterior uterine and cervical wall. The baby was delivered by transverse cervicotomy caudally of the placenta. The placenta was left in situ. The patient then got prophylactic embolization of the uterine arteries to prevent further severe hemorrhage. 48 h later, ultrasound showed a floating, avascular placenta within a poor echogenic fluid-filled cervical space as well as macrohematuria. After re-laparotomy and cervicotomy at the same day, the placenta was completely and easily evacuated. A bladder injury was recognized and closed. We performed a cervical internal os plasty by inverting the cervical lips and suturing their distal ends on the proximal cervical tissue, resulting in complete bleeding cessation. Although, the patient got 8 erythrocyte concentrates at all, she was always in a stable condition without hemorrhagic shock.

This case demonstrates for the first time a live-birth with uterus-conserving management in CEP.

**Supplementary Information:**

The online version contains supplementary material available at 10.1186/s12884-023-05951-5.

## Background

 5% of all ectopic pregnancies have a non-tubal localization and 15% of non-tubal pregnancies are located in the cervix [1]. A cervical ectopic pregnancy (CEP) is defined as nidation within the endocervix belong the internal os [2]. Risk factors for CEP are previous operations of the uterus [2], ART (artificial reproductive techniques) and Ashermans syndrome [3]. The main complication is life-threatening bleeding due to abnormal vascularization within the cervix and the impossibility of cervical tissue to seal the cervicoplacental vessels by contraction after the evacuation of the placenta [2]. An extensive review of the literature showed that there was no report of a case resulting in a live birth and uterus-conserving therapy. Standards of care are early termination of pregnancy by methotrexate [4], uterine artery embolization (UAE) [5], curettage [6] or office hysteroscopy [7], intracervical balloon placement [2] and intracervical injection of vasopressin [8].

Here, we report the first case of a live birth after complete cervical implantation including uterus-conserving management.

## Case presentation

**Fig. 1:**
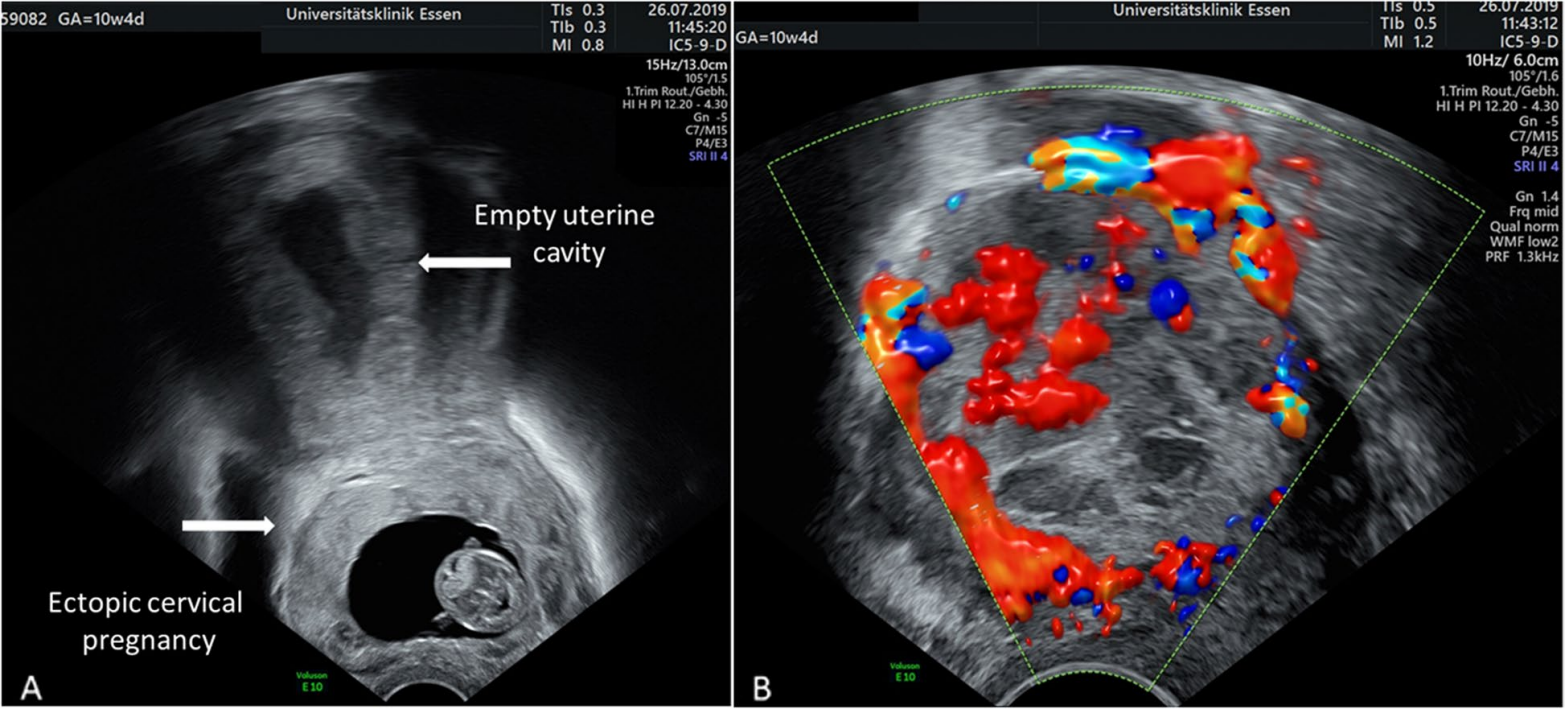
** A **Cervical ectopic pregnancy with the gestational sac was located between the internal and external cervical os. **B** Strong vascularization and lacunae in vaginal Doppler sonography

A 37-old III gravida II para with two previous cesarean sections (CS) presented in 7 + 3 weeks of pregnancy with CEP. Sonographic criteria for complete cervical implantation were present: the uterine cavity was empty and the gestational sac was located between the internal and external cervical os (Fig. 1A). There was no adherence to a previous cesarean scar niche. Strong vascularization and lacunae were present (Fig. 1B). After extensive information about the risks (severe bleeding and the loss of her uterus at any time of pregnancy), the patient decided not to terminate the pregnancy for ethical reasons.

At the 12th week of pregnancy, we performed a cerclage to avoid cervical distention by the expanding placenta. Since the natural barrier of the intact cervical channel containing cervical mucus was completely replaced by placental mass, we hypothesized an increased risk of ascending infections. For prevention, the patient presented weekly for clinical examination, determination of blood parameters (leucocytes and c-reactive protein), vaginal smear and cleaning of the vagina with 30 ml Lavasorb® (polyhexanid and macrogol). Until 26 + 0 weeks of gestation, pregnancy was uneventful and without vaginal bleeding episodes. In 29 + 5 weeks of gestation, corticoids were given for prevention of respiratory distress syndrome in case of preterm delivery. Due to missing experience with CEP management and to avoid an emergency operation, we decided on delivery in 30 + 0 weeks of gestation when the patient additionally reported unspecific abdominal pain.

**Fig. 2:**
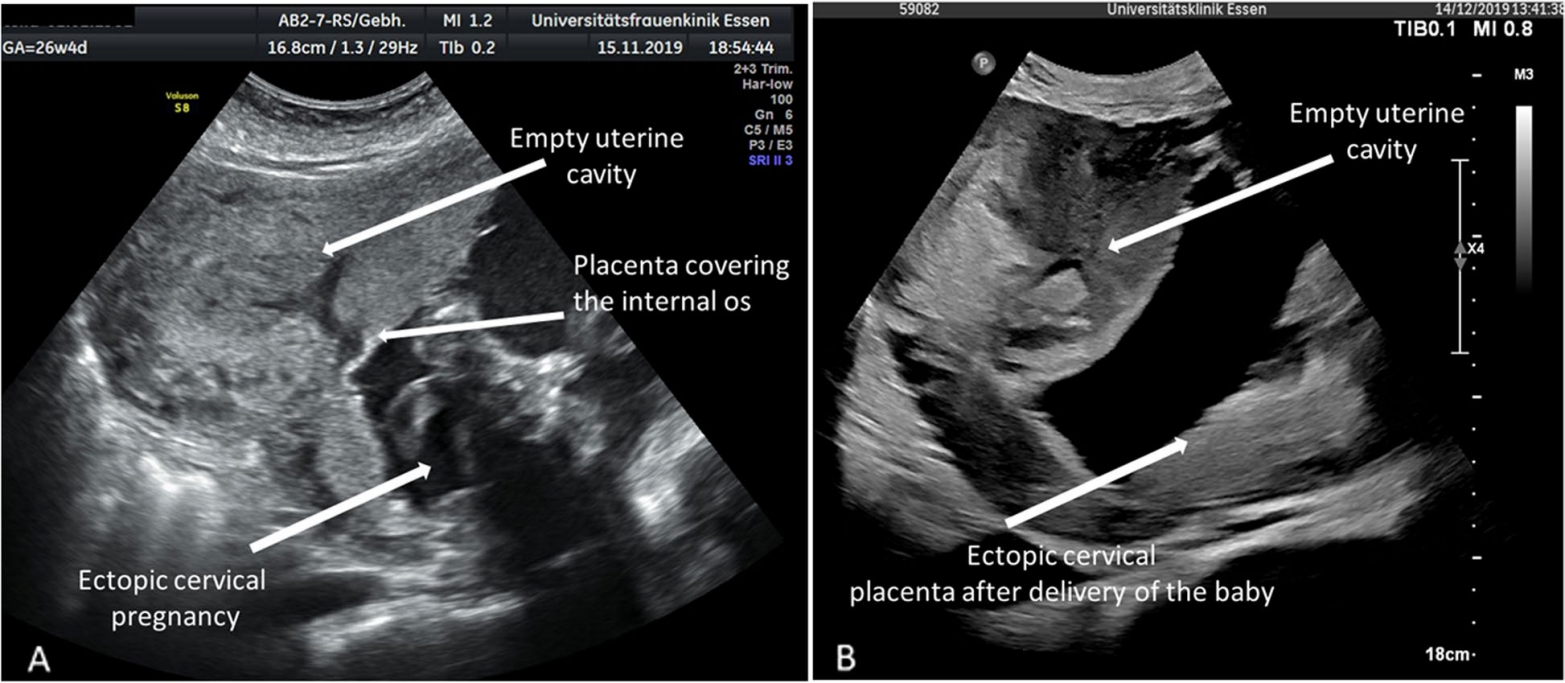
** A** Cervical cavity was covered by the placenta including the internal os in pregnancy. **B** Cervical cavity was covered by the placenta including the internal os after delivery of the baby

We planned to leave the placenta in situ after delivery of the baby as a known efficacious procedure in cases of an abnormally invasive placenta (AIP) (Senthiles et al., 2018). Since the cervix uteri has no comparable muscle tissue like the corpus uteri that is able to produce contractions resulting in the delivery of the baby, we opted for a planned caesarean section. However, a fundal incision for fetal delivery was not possible since the whole cervical cavity was covered by the placenta including the internal os (Fig. 2A, 5 left). Only the most distal cervical tissue was free of placental attachment. Consecutively, we planned fetal delivery per cervicotomy followed by UAE after the baby´s delivery. For procedure planning an MRI with MR angiography was performed.

**Fig. 3:**
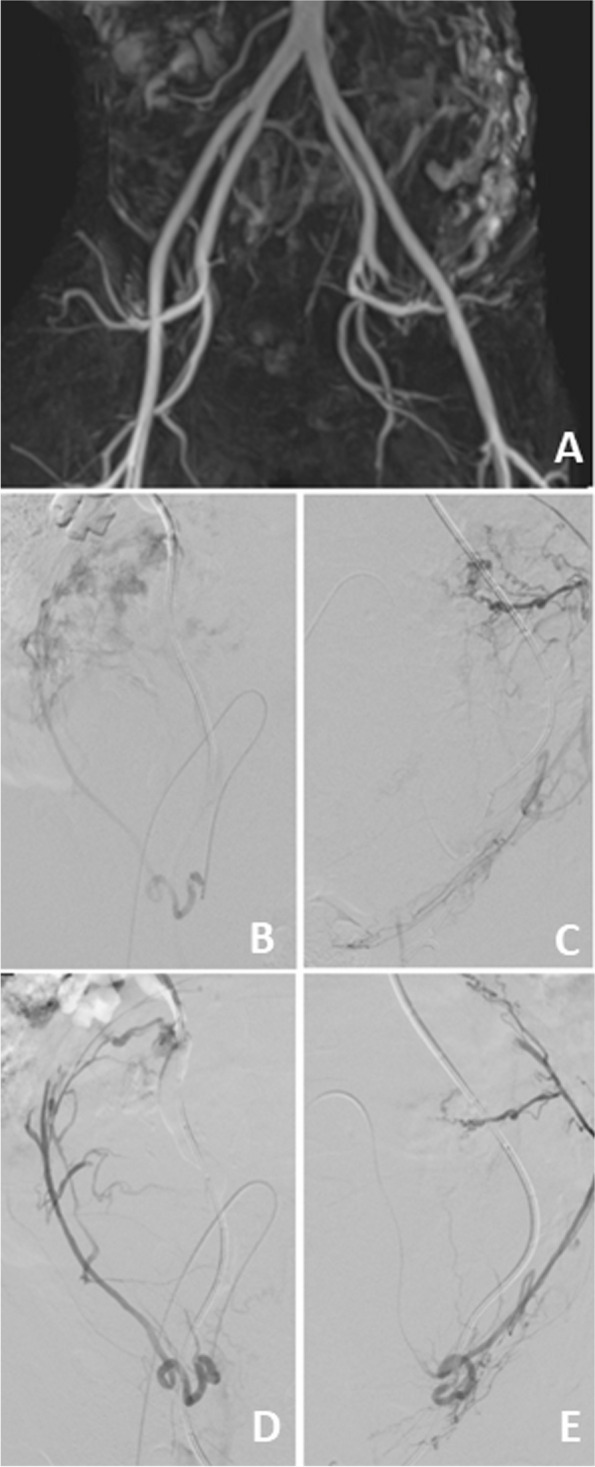
Anatomic overview of pelvic arteries with 3D-MR-angiography as navigation guide for DAS (**A**). Catheter placement in right and left uterine arteries before (**B**, **C**) and after (**D**, **E**) UAE showing reduced perfusion to the uterus

Starting the operation with spinal anesthesia, ureteral stents were placed to prevent ureteral damage in case of an unplanned intrapartum hysterectomy. After transverse laparotomy, the urinary bladder was sharply dissected from the anterior uterine and cervical wall. Intra-operative sonography enabled us to identify the scarce placenta-free cervical tissue near the external os. The baby was delivered by transverse cervicotomy caudally of the placenta using Ligasure® scissors (Video S1). A male newborn with a weight of 1620 g was born. APGAR score was 8/8/9. Afterward, the cervical incision was closed rapidly with a continuous non-barbed suture using Vicryl CT-1. Cervicotomy was accompanied by strong bleeding. The patient got general anesthesia during this episode and received 2 red blood packages. After the closure of the uterus, the bleeding stopped immediately and the abdomen was closed. The patient remained in general anesthesia for UAE. During the angiography, the uterine artery was catheterized sequentially on both sides using a 4 F catheter (Glidecath, Terumo) in vertebral configuration. A coaxially introduced microcatheter (Renegade Hi-Flo, Boston Scientific) was advanced into the uterine artery on both sides, and calibrated microspheres (700–900 μm and 900–1200 μm, 1ml of each, Embosphere, Merit Medical) were given until visual reduction of placenta perfusion was achieved (Fig. 3A-E).

**Fig. 4:**
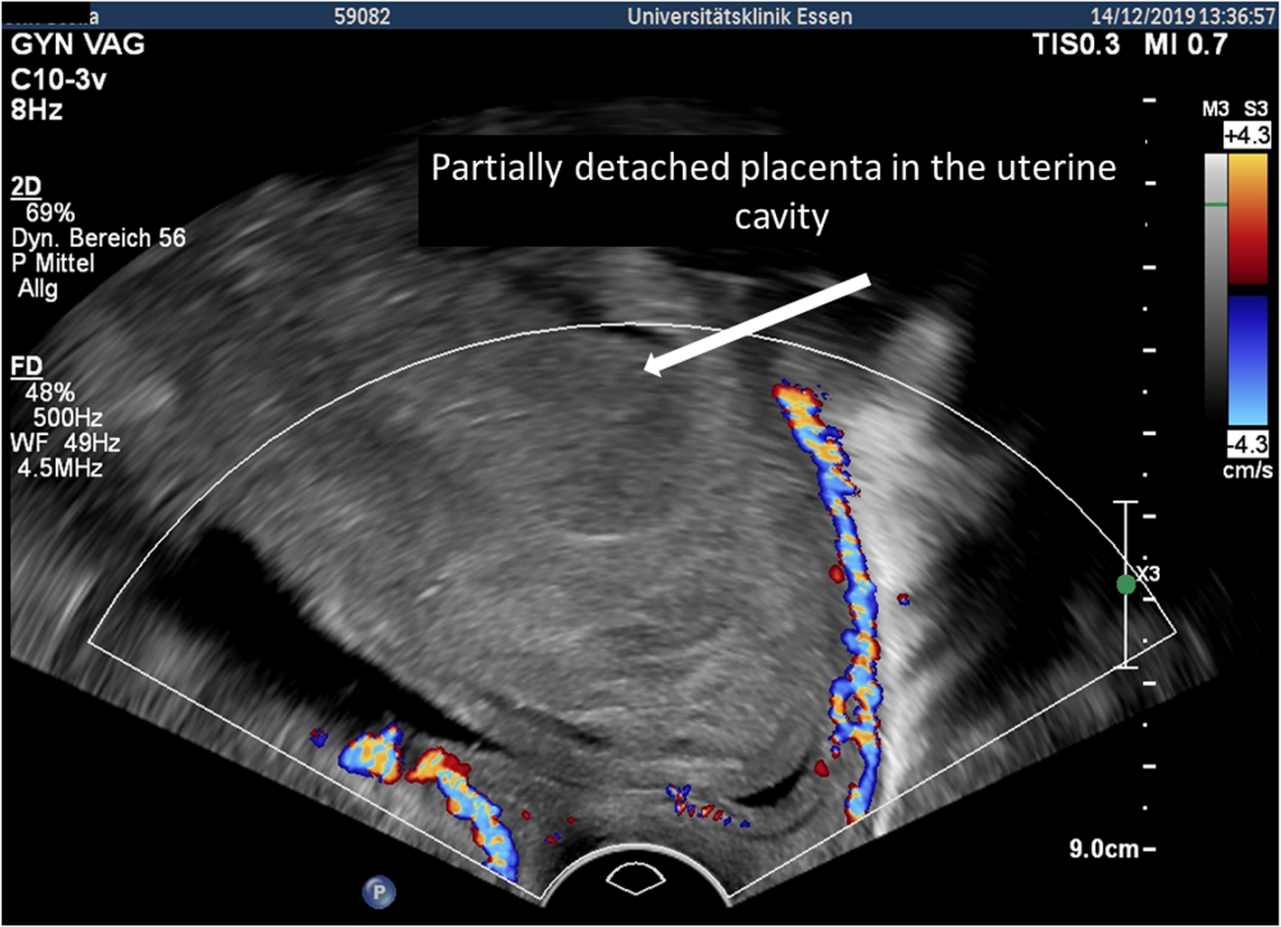
Partially detachted placenta after delivery of the baby and uterine artery embolization

**Fig. 5:**
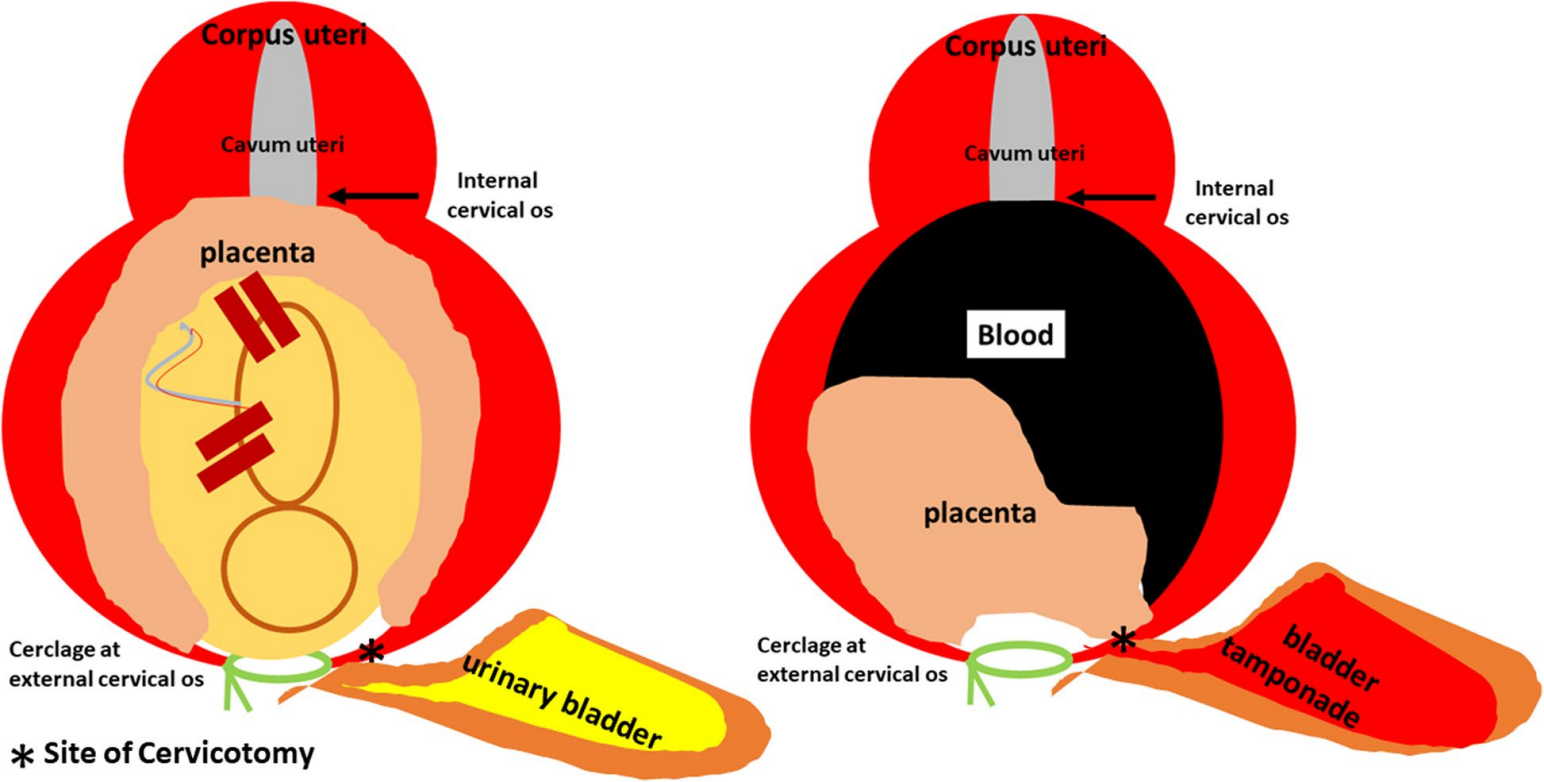
Left: Cervical cavity covered by the placenta including the internal os in pregnancy. Right: Injury of the bladder resulting in macrohematuria and urine diffusion in the cervical cavity with partial detachment of the placenta

Figure 2B shows the cervix with the placenta in situ after the delivery of the baby. Afterward, the patient developed progredient macrohematuria with decreasing hemoglobin requiring the transfusion of 2 additional red blood packages within 48 h. Ultrasound showed a floating, avascular placenta within a poor echogenic fluid-filled cervical space above the placental attachment (Fig. 4). We hypothesized a partial detachment of the placenta (Fig. 5 right) and decided on placental delivery via re-laparotomy. The cerclage was removed.

**Fig. 6:**
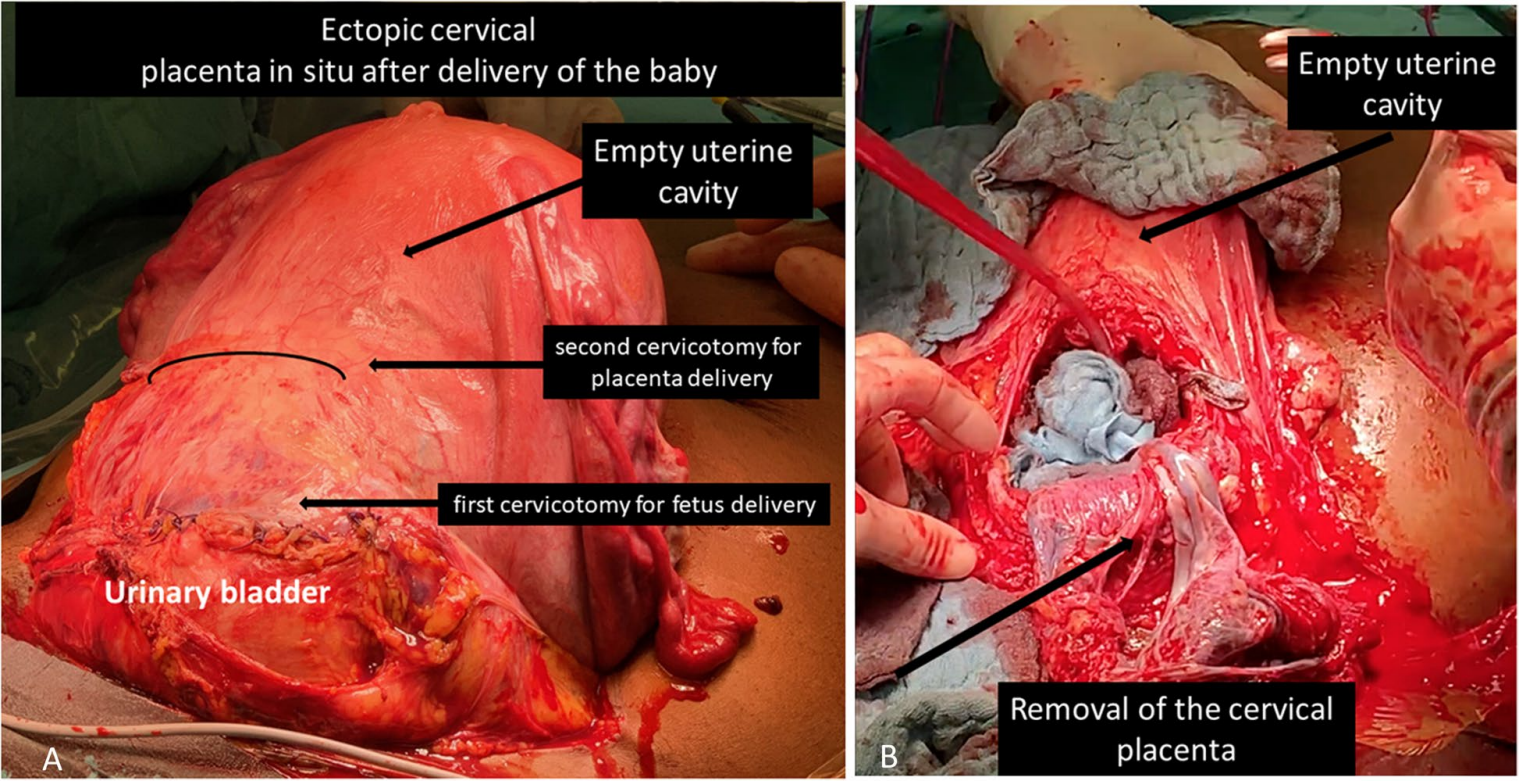
** A** Illustration of ultrasound guided decision of the opening site of the anterior cervical wall. **B** Removal of the cervical placenta

**Fig. 7:**
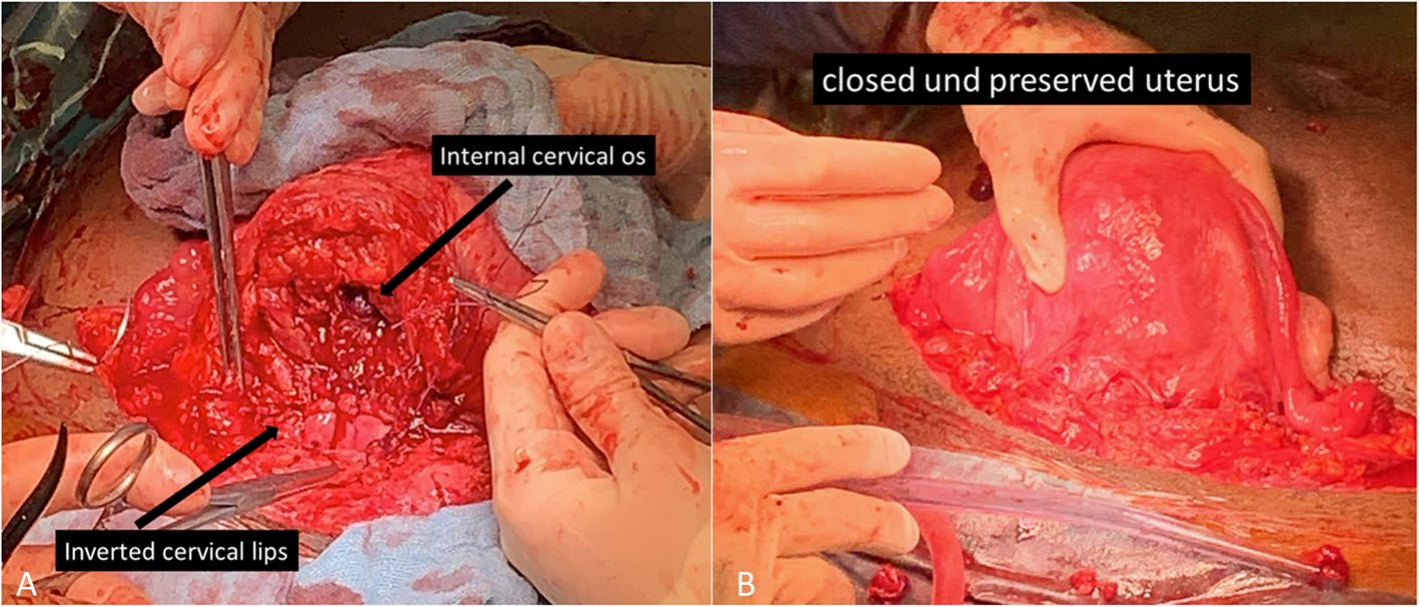
** A** Cervical internal os plasty. **B** Closed and preserved uterus

After re-laparotomy, intraoperative sonography was used to identify the placental position and decision for an opening site of the anterior cervical wall (Fig. 6A). Severe hemorrhage occurred after cervicotomy indicating that blood was inside the cervix. Since vaginal bleeding never occurred before, we suppose that the distal part of the placenta was still adherent to the cervical wall while the cranial part of the placenta already was detached (Figs. 4 and 5 right). The placenta was removed rapidly, but easily (Fig. 6B). The placental weight was 199 g. A bladder injury was identified and closed by the urologist. The bladder injury explains the macrohematuria since the leakage connected the area above the detached placenta and the urinary bladder (Fig. 5 right) After the closure of the bladder injury, we performed a so-called cervical internal os plasty by inverting the cervical lips and suturing their distal ends on the proximal cervical tissue as described by Huang et al., 2019 [9] (Fig. 7A). Using this technique, the bleeding stopped completely. The cervicotomy was closed by single stitches using Vicryl CT-1 (Fig. 7B). The patient once again received 4 red blood packages during the second operation. Postoperative recovery was uneventful and the patient was discharged after 14 days.

## Discussion and conclusion

Available literature includes many case reports about CEP, whereas only one reported case resulted in a live birth [10] in 28 weeks of gestation followed by an emergency hysterectomy.

CEP treatment options in the first trimester include methotrexate (systemic therapy [4] or transvaginal injection [11], UAE in combination with methotrexate infusion [5], UAE followed by curettage [6] or office hysteroscopy [7], balloon application [12] and intracervical injection of vasopressin [8].

Only very few cases describe second-trimester CEP management [13, 14]. In all cases, pregnancy termination was induced after diagnosis. In one case, maternal hydronephrosis due to a kinked ureter because of the bulged cervix led to the diagnosis of a 21–22 weeks CEP. The case was treated by laparotomy as an attempt to conserve the uterus, but severe hemorrhage after removal of the placenta required emergency hysterectomy [15]. A further case of late first-trimester CEP was treated by UAE and methotrexate. 14 days after, severe hemorrhage occurred, but was stopped by curettage [16]. In summary, the reported cases of advanced CEP all were associated with severe hemorrhage after placental detachment.

In contrast, live birth is published in cases of isthmocervical pregnancies [17, 18]. However, nearly all patients needed an emergency hysterectomy. Histological examen showed AIP (abnormally invasive placenta) [17, 18]. We recently published a case of isthmocervical pregnancy with severe hemorrhage in the second trimester [19]. In this case, embolization of cervical branches of the uterine artery was performed in the 19th week of pregnancy resulting in bleeding arrest for 10 weeks. In the 29th week of pregnancy, severe hemorrhage occurred again and a hysterectomy was performed after the fundal delivery of the preterm but healthy baby. 

Concerning the pathogenesis of AIP, it is well known that the decidua plays a crucial role in the control of trophoblast invasion and the associated spiral artery remodeling [20]. AIP always is a consequence of an endometrial defect [20]. If the decidua is completely absent, as is the case in the endocervix, CEP will result per definition always in AIP, characterized by excessive vascularization [20]. In contrast to a CSP (cesarean scar pregnancy), where there is a mechanical defect in anterior uterine wall, the placenta does not overwhelm the surface and does not penetrate the cervical wall in CEP, allowing a manual or instrumental evacuation. Since the cervical wall is not able to perform contractions like the myometrium, vessel sealing is strongly impaired after placental detachment, resulting in a life-threatening hemorrhage. On the other side, the missing ability of the cervix to cause contractions may explain the lack of antenatal bleeding in our and several of the published cases. Therefore, CEP is strongly characterized by severe bleeding after placental detachment but not always before placental delivery.

In conclusion, one main first goal of CEP management is LISA (leaving the placenta in situ approach). As a second step, bleeding prevention procedures should be performed before the placenta will be detached. Here, we performed embolization of the cervical branches of the uterine artery after the delivery of the baby and the cervical internal os plasty after the complete detachment of the placenta.

A study of 29 cases showed the high efficiency of the UAE in preventing life-threatening bleeding in CEP and postpartum AIP [21]. Several cases of efficacious prophylactic UAE followed by curettage in the first-trimester CEP have been reported [6]. However, UAE in advanced gestational age was still associated with severe bleeding [15].

We also hypothesized that the uncomplicated pregnancy course was a result of a very intensive care and infection prevention approach in our patient. The cerclage itself contributed to cervical closure by prevention of cervical dilatation through the growing placenta.

In summary, the following approaches enabled a live birth and uterus-conserving management in our patient:

1. Cerclage
2. Prevention of ascending infection
3. Fetal delivery outside of the placental bed
4. LISA (leaving the placenta in situ approach)
5. UAE (cervical branches)
6. Re-laparotomy with rapid removal of the placenta and cervical internal os plasty

Taking all these approaches together, successful pregnancy and life birth with preservation of the uterus as well as fertility was possible.

### Supplementary Information

**Additional file 1: Video S1.** Delivery of the baby in complete cervical pregnancy with an empty cavum uteri.

## Data Availability

All data generated or analysed during this study are included in this published article.

## Abbreviations

AIP: Abnormally invasive placenta
ART: Artificial reproductive technique
CEP: Cervical ectopic regnancy
CS: Cesarean section
CSP: Cesarean scar pregnancy
DAS: Digital subtraction angiography
LISA: Leaving the placenta in situ approach
UAE: Uterine artery embolization
